# Electroacupuncture Alleviates Surgical Trauma-Induced Hypothalamus Pituitary Adrenal Axis Hyperactivity Via microRNA-142

**DOI:** 10.3389/fnmol.2017.00308

**Published:** 2017-09-27

**Authors:** Jing Zhu, Zhejun Chen, Zehui Meng, Minda Ju, Mizhen Zhang, Gencheng Wu, Haidong Guo, Zhanzhuang Tian

**Affiliations:** ^1^Department of Anatomy, School of Basic Medicine, Shanghai University of Traditional Chinese Medicine, Shanghai, China; ^2^Department of Nephrology, Molecular Cell Laboratory for Kidney Disease, Ren Ji Hospital, School of Medicine, Shanghai Jiao Tong University, Shanghai, China; ^3^Department of Integrative Medicine and Neurobiology, State Key Laboratory of Medical Neurobiology, Collaborative Innovation Center for Brain Science, Institute of Acupuncture Research, WHO Collaborating Center for Traditional Medicine, The Institutes of Integrative Medicine of Fudan University, Fudan University, Shanghai, China

**Keywords:** hypothalamus pituitary adrenal axis, corticotrophin releasing hormone, microRNA-142, electroacupuncture, hepatectomy

## Abstract

Electroacupuncture (EA) could improve the hyperactivity of the hypothalamus pituitary adrenal (HPA) axis induced by hepatectomy. However, its underlying mechanism still remains largely unclear. Here, we found that hypothalamic corticotrophin releasing hormone (CRH) modulates the function of the HPA axis, while hepatectomy induced an HPA axis disorder and EA application could regulate the hypothalamic CRH. We first demonstrated that microRNAs (miRNAs) target on CRH via bioinformatics analysis and screened them in the primary hypothalamic neurons. MicroR-142 (miR-142) and miR-376c were identified to inhibit CRH at the mRNA and protein levels, and a dual luciferase reporter assay confirmed their binding to the 3′-untranslated regions (3′-UTR) of CRH. Further analyses revealed a decrease in hypothalamic miR-142 expression in the hepatectomy rats and an increase in miR-142 and miR-376c after EA intervention. Importantly, the improvement effect of EA on the HPA axis regulatory function in hepatectomy rats was blocked by miR-142 antagomir. Our findings illustrated that EA could up-regulate hypothalamic miR-142 expression and decrease the CRH level to alleviate the hyperactivity of the HPA axis induced by hepatectomy.

## Introduction

Surgery or severe trauma induces the dysfunction of the neurogenic, immune and endocrine systems (Aller et al., 2013), resulting in immunosuppression, aprosexia and other stress reactions (Morrison et al., 1996), always with a poor prognosis. Although some treatments have contributed to improving these reactions (Marik and Flemmer, 2012), it is still hard to rectify trauma-induced homeostasis disorder. The hypothalamus pituitary adrenal (HPA) axis plays an inevitable role in controlling the stress reaction, especially those induced by surgery and severe injury (Gibbison et al., 2013). Electroacupuncture (EA) has been proven to normalize the HPA axis dysfunction during surgery (Zhu et al., 2016), but its potential mechanism still needs to be elucidated.

The paraventricular nucleus (PVN) in the hypothalamus, adenohypophysis and the cortex of the adrenal glands contribute to the HPA axis. Corticotrophin releasing hormone (CRH), mostly synthesized and secreted from the parvocellular of the PVN as the initiation part of the HPA axis, stimulates adrenocorticotropic hormone (ACTH) synthesis and secretion from the adenohypophysis to the blood, and promotes glucocorticoid hormone (GC, human: cortisol; Rodent: corticosterone, CORT) synthesis and secretion. Nevertheless, there is surprisingly little known about the role of hypothalamic CRH in severe injury or surgical trauma. Moreover, the neural mechanism by which surgery produces HPA axis disorder is unclear.

MicroRNAs (miRNAs) are small noncoding RNAs that can inhibit the function of gene expression via binding to the 3′-untranslated regions (3′-UTR) of the gene. There are abundant data about the function of miRNAs in the development (Chen and Qin, 2015) or disease of the central nervous system. MiRNAs were reported to play a critical role in depression (Serafini et al., 2014) while the HPA axis was also implicated in the depression (Schatzberg et al., 2014), the positive association between miRNAs and HPA axis deserved to be elucidated. Pituitary MicroR-449a (miR-449a) impairment induced a decrease in the level of CRH receptor 1 (CRHR1) level in low-birth-weight rats (Nemoto et al., 2015), and miR-449a was involved in the GC induced CRHR1 downregulation (Nemoto et al., 2013). GC function was related to miR-338 (Zhang et al., 2016) and miR-433 (Smith et al., 2016) in osteoclast formation. Hypothalamic miRNAs reportedly played a critical role in energy balance (Schneeberger et al., 2015). However, there was no evidence concerning hypothalamic miRNAs in the modulation of the HPA axis function. Our previous study proved that EA could attenuate the hyperactivity of the HPA axis in hepatectomy rats (Zhu et al., 2016), but whether miRNAs were involved in the EA regulation effect of HPA axis in hepatectomy was still a question.

The present study thus examines the effects of EA on the adjustment of hypothalamic CRH, and screens the miRNAs targeting CRH in the hypothalamus in order to clarify the underlying mechanism of EA in modulating the function of the HPA axis in the hepatectomy rats.

## Materials and Methods

### Animals

Adult, male SD rats (200 ± 10 g) purchased from the Experiment Animal Center of the Chinese Academy of Sciences (Shanghai, China) were housed five per cage in a 12 h light/dark normal cycle at 22°C with food and water *ad libitum*. All experiments were done in accordance with the National Institute of Health Guide for the Care and Use of Laboratory Animals (NIH Publication No. 23-80, revised 1996) and they were approved by the research ethical standards for the care and use of animals at Fudan University. Additionally, care was taken to minimize the number of animal used in each experiment and their suffering.

### Experimental Design

#### Experiment 1

Rats were divided into the control, hepatectomy, sham-EA+hepatectomy (SEA) and EA+hepatectomy (EA) groups. Rats in the four groups were sacrificed at 4 h and 1 day immediately after surgery based on previous study (Zhu et al., 2016). There were seven rats in each group per time point. They were sacrificed by decapitation, and their sera and hypothalamus tissues were kept at −80°C for further assessment. Additionally, there were five rats in each group per time point for immunohistochemistry.

#### Experiment 2

A drug delivery system from RWD Life Science (RWD, 62037, 62137, 62237, Shenzhen, China) was fixed into the brain of rats 10 days before drug application at 1.5 mm rostral, 0.4 mm lateral, 7.8 mm ventral to bregma (Li T. T. et al., 2014), the injection site was shown in Supplementary Figure S1. Then, the rats were divided into the control, hepatectomy, hepatectomy+scramble siRNA (siRNA) and hepatectomy+siCRH (siCRH) groups. Each side was given 2 μl containing 500 pmol siCRH or scramble siRNA, and the injection velocity was 0.5 μl/min (Huang L. et al., 2015). The drug was injected 4 days and 1 day before surgery between 8:00 and 10:00 AM. There were five rats in each group per time point. They were sacrificed by decapitation, and their sera were kept for detection.

#### Experiment 3

A drug delivery system was fixed similar to the Experiment 2. Then the rats were divided into the control+scramble, hepatectomy+scramble, control+miR-142, hepatectomy+miR-142, control+miR-376c and hepatectomy+miR-376c groups. Each side was given 2.5 μl of 40 nmol/ml miRNA agomir or scramble miRNA agomir. The injection velocity was 0.5 μl/min, and the injection was performed 4 days and 1 day before surgery between 8:00 and 10:00 AM (Huang L. G. et al., 2015). There were six rats in each group and their sera were harvested for hormone detection.

#### Experiment 4

A drug delivery system was fixed as in Experiment 2. Then, the rats were divided into the scramble, hepatectomy+scramble, hepatectomy+scramble+EA, hepatectomy+miR-142+EA and hepatectomy+miR-376c+EA groups. Each side was given 2.5 μl of 40 nmol/ml miRNA antagomir or scramble miRNA antagomir. The injection velocity was 0.5 μl/min, and the injection was performed at 4 days and 1 day before surgery at between 8:00 and 10:00 AM. There were six rats in each group, and their sera were harvested for detection.

### Surgery

Rats were given a 10% partial hepatectomy as described before (Zhu et al., 2016). Briefly, after anesthesia with pentobarbital sodium (30 mg/kg), rats were given a surgical incision, which was approximately 7 cm long from the cartilago ensiformis to the symphysis pubis along the linea alba. The abdominal cavity was opened, and 10% of the liver was removed from the right lobe. Then, the bleeding was stemmed and the abdominal cavity was closed. All surgeries were done under aseptic conditions and between 8:00 and 10:00 AM.

### Electroacupuncture

EA application was performed as described before (Zhu et al., 2016). Two stainless steel acupuncture needles 0.25 mm in diameter were inserted at a depth of 10 mm into the “Zu San Li” (ST36, located in the posterolateral knee joint, approximately 0.5 cm below the capitulum fibulae) and “San Yin Jiao” (SP6, at the superior border of the media malleolus, between the posterior border of the tibia and anterior border of the Achilles tendon), respectively (Supplementary Figure S2). The needle handle for EA application was connected to the output of a HANS Acupoint Nerve Stimulator (LH202H, Beijing, China), and the stimulation lasted for 30 min (8:00–10:00 AM) at an intensity of 2 mA with alternating strings of dense-sparse frequencies (2 Hz for 1.05 s and 15 Hz for 2.85 s, alternating). However, only acupuncture needle insertion into ST36 and SP6 was done for sham-EA application rats.

### Cell Culture

Rats at P0 age were sacrificed by decapitation. Then, their brains were extracted into Hank’s Balanced Salt Solution (HBSS, 14175095, Thermo Fisher, CA, USA), and their hypothalamus tissues were separated. After visible blood vessels were dislodged, the hypothalamus tissues were digested with 0.25% Trypsin-EDTA (25200056, Gibco, CA, USA) at room temperature for 20 min, during which the cell suspension was gently broken up via a Pasteur pipette. Then, the cells were harvested via filtration and centrifugation. The cells were cultured in Neurobasal Medium (21102049, Thermo Fisher, CA, USA) with 2% B27 (17504-044, Thermo Fisher, CA, USA) and 10% fetal bovine serum (FBS, 10082147, Thermo Fisher, CA, USA). Then the cells were inoculated in 24-well plates at 7 × 10^5^ under 37°C and a 5% CO_2_ atmosphere.

Additionally, HEK 293T cells were cultured in Dulbecco’s Modified Eagle’s Medium/Nutrient Mixture F12 (DMEM/F12, SH30023.01, HyClone, UT, USA) with 10% FBS.

### SiRNA and miRNA Transfection

All siRNA (Supplementary Table S1) and miRNA used in this experiment were synthesized by Ribo (Guangzhou, China). Approximately 1 × 10^5^ hypothalamus neurons were incubated in 500 μl culture medium with a mixture of 5 μl of 10× Ribo FECT CP Buffer (C10511-1, Guangzhou, China), 1.25 μl of 20 μM siRNA or miRNA and 5 μl Ribo FECT CP Reagent for 48 h at 37°C, in 5% CO_2_ atmosphere.

### Methylthiazolyldiphenyl-Tetrazolium Bromide Assay

Cell viability was detected via a colorimetric assay using a yellow tetrazolium, methylthiazolyldiphenyl-tetrazolium bromide (MTT, C0009, Beyotime, China). The cells were incubated with 5 mg/ml of MTT solution for 4 h, and then formazan for 4 h at 37°C. The absorbance at 570 nm was determined.

### Plasmid Construction and Luciferase Assay

MiRNA target prediction programs (TargetScan and MiRanda) were used to screen the miRNAs targeting the 3′-UTR of CRH and their binding sites. The 3′-UTR of CRH was amplified using rat genomic DNA as a template. The PCR products were subcloned into the region directly downstream of the stop codon in the luciferase gene in the luciferase reporter vector to generate the p-Luc-UTR reporter plasmid (primers was in Supplementary Table S2). Overlap PCR was used to construct the 3′-UTR mutant reporter plasmid.

The sequences of the wild-type and mutant 3′-UTR were confirmed by sequencing. For the luciferase assay, HEK 293T cells were cultured in 24-well plates and co-transfected with a mixture of 120 ng of p-Luc-UTR, 20 pmol of the miRNA agomir, and 20 ng of the Renilla luciferase vector pRL-CMV (Promega, Madison, WI, USA) using FuGENE HD Transfection Reagent (E2311, Promega, Madison, WI, USA). The firefly and Renilla luciferase activities were measured 2 days after the transfection via a dual-luciferase reporter assay system (E1910, Promega, Madison, WI, USA) from the cell lysates.

### Real-Time Polymerase Chain Reaction

Total RNA was extracted using TRIzol Reagent (15596018, Thermo Fisher, Carlsbad, CA, USA) according to the manufacturer’s instructions. The quality and quantity of RNA in the samples was determined using a NanoDrop spectrophotometer (ND-2000, Infigen Biotechnology Inc., Industry, CA, USA). Approximately 2000 ng of total RNA were reverse transcribed via the Go-Script Reverse Transcription System (Promega, Madison, WI, USA), and the other 2000 ng of total RNA were reverse transcribed with the corresponding stem-loop RT primer (Ribo Bio, Guangzhou, China) for miRNA detection. The mRNA primers (Supplementary Table S3) and miRNA primers were purchased from Ribo Bio.

Real-time polymerase chain reaction (Real-time PCR) was conducted using an iQ 5 real-time PCR detection system (Bio-Rad, Hercules, CA, USA). The reaction volume included 10 μl of SYBR Green Real Master Mix (Promega, Madison, WI, USA), 1.6 μl of the primer mixture (200 nM), 1.6 μl of the cDNA template and 6.8 μl of diethyl pyrocarbonate (DEPC) H_2_O. The thermocycler program was as follows: 5 min at 95°C; 40 cycles of 45 sat 94°C, 1 min at 59°C, and 1 min at 72°C; and 1 min at 72°C. The purity of the PCR products was confirmed via melting curve analysis. All reactions were run in triplicate. The relative expression of miRNA and mRNA was calculated using the comparative method with U6 or glyceraldehyde-3-phosphate dehydrogenase (GAPDH) as the reference gene, respectively.

### Western Blot

Total proteins were extracted using the Total Protein Extraction Kit (P0013B, Beyotime, China). Then, the liquid supernatant was denatured at 100°C for 10 min in loading buffer (161-0747, Bio-Rad, Hercules, CA, USA), and equal amounts of protein (40 μg) were separated on a 10% SDS-PAGE gel. The CRH protein level was discovered via western blot (WB) using rabbit polyclonal anti-CRH antibody (ab8901, 1:1000, Abcam, Cambridge, UK). The blot was incubated with the antibody at 4°C overnight and successively with a horseradish peroxidase (HRP)—conjugated goat anti-rabbit IgG (H + L) for 2 h at room temperature after washing with tris buffered saline and tween 20 (TBST). Immunoreactivity bands were detected using the Immobilon Western Chemiluminescent HRP Substrate (P90720, Millipore, Darmstadt, Germany) with an Image-Quant LAS 4000 Mini system (GE Healthcare, Buckinghamshire, UK). The protein expression was quantified using Quantity One software (Bio-Rad, Hercules, CA, USA) and normalized against the GAPDH (10494-1-AP, 1:10,000, Proteintech, IL, USA) control.

### Immunofluorescence

Rats were perfused with phosphate buffered saline (PBS, pH = 7.4) followed by 4% paraformaldehyde (PFA) in 0.1 M PBS (pH = 7.4). Their brains were harvested and left in 4% PFA overnight. The rat brains were cryoprotected using 20% sucrose in 4% PFA and successively by 30% sucrose in 0.1 M PBS. After their sedimentation, the brains were cut at a thickness of 30 μm for each section. The slices including PVN area (located at Interaural 7.56 mm, Bregma −1.44 mm ~ Interaural 6.96 mm, Bregma −2.04 mm according to the *The rat brain in stereotaxic coordinates 5th*) were stored in a cryoprotectant solution in −20°C. These sections for each group were washed in 0.01 M PBS and incubated in Immunol Staining Blocking Buffer (P0102, Beyotime, China) at 37°C for 1 h and then with the sheep polyclonal anti-CRH antibody (NB110-81721, 1:100, Novus bio, CO, USA) for 48 h at 4°C. They were then incubated with Donkey anti-Sheep IgG (H + L) Cross Adsorbed Secondary antibody conjugated to Alexa Fluor 488 (A11015, 1:800, Thermo Fisher Scientific, Rockville, MD, USA). All sections were observed under a fluorescence microscope (Leica, Germany).

### *In Situ* Hybridization

Hypothalamus PVN miR-142 and miR-376c expression was detected by *in situ* hybridization (ISH) analysis. The oligonucleotide probe to rno-miR-142 was 5′-AGTAG TGCTT TCTAC TTTAT G-3′, and the probe to rno-miR-376c was 5′-ACGTG AAATT TCCTC TATGT T-3′ (Boster Inc., Wuhan, China). The rat brains were fixed in 4% PFA, embedded, and sectioned. The miR-142 and miR-376c expression in each brain slice was detected using an enhanced sensitive ISH detection kit (Boster Inc., Wuhan, China) according to the manufacturer’s instructions. Then, the positive miRNA expression of PVN was detected under an optical microscope.

### Enzyme-Linked Immunosorbent Assay

Serum CRH, ACTH, CORT and CRH in the culture medium were detected using (enzyme-linked immunosorbent assay, ELISA) kits. Briefly, a concentration gradient of a standard working solution of each sample was prepared. Biotin binding substrate was added to each sample and incubated in a humidified box for 2 h at room temperature. After washing three times, HRP binding substrate was added to each sample, and incubated for 1 h. Then, 3,3,5′,5′-tetramethyl benzidine (TMB) was added for 10 min without light, and stop buffer was added. The concentration was calculated from the optical density (OD) value at 450 nm.

### Statistical Analysis

The comparisons among groups and the corresponding controls were performed with SPSS 17.0 (IBM, USA) using Student’s *t* test or analysis of variance (ANOVA) followed by a Tukey post-test. Error bars are representative of the standard derivation (SD) values. *P* values of 0.05 or less were considered significant.

## Results

### EA Attenuated the HPA Axis Hyperactivity Induced by Hepatectomy

**Figure 1:**
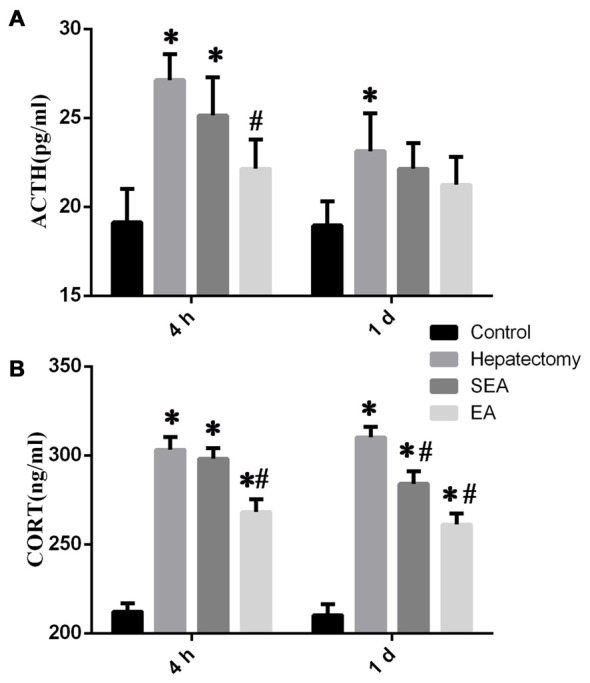
The level of stress-related hormones determined by enzyme-linked immunosorbent assay (ELISA) in the peripheral blood. Blood adrenocorticotropic hormone (ACTH; **A**) and CORT **(B)** levels in the control, hepatectomy, SEA and electroacupuncture (EA) groups at 4 h and 1 day after hepatectomy. The data are presented as the mean ± SD (*n* = 7) *vs. control group (*p* < 0.05); ^#^vs. hepatectomy group (*p* < 0.05).

Serum ACTH level in the hepatectomy group was up-regulated compared with that in the control group at 4 h (*p* < 0.05) and 1 day (*p* < 0.01) after hepatectomy (Figure 1A). Compared with that of the hepatectomy group, the serum ACTH level in the EA group was decreased significantly at 4 h (*p* < 0.01) after hepatectomy. Meanwhile, compared with that of the control group, the serum CORT level in the hepatectomy group was increased at 4 h (*p* < 0.05) and 1 day (*p* < 0.01). Compared with the hepatectomy group, the serum CORT level in the EA group was decreased at 4 h (*p* < 0.01) and 1 day (*p* < 0.01) after hepatectomy (Figure 1B).

**Figure 2:**
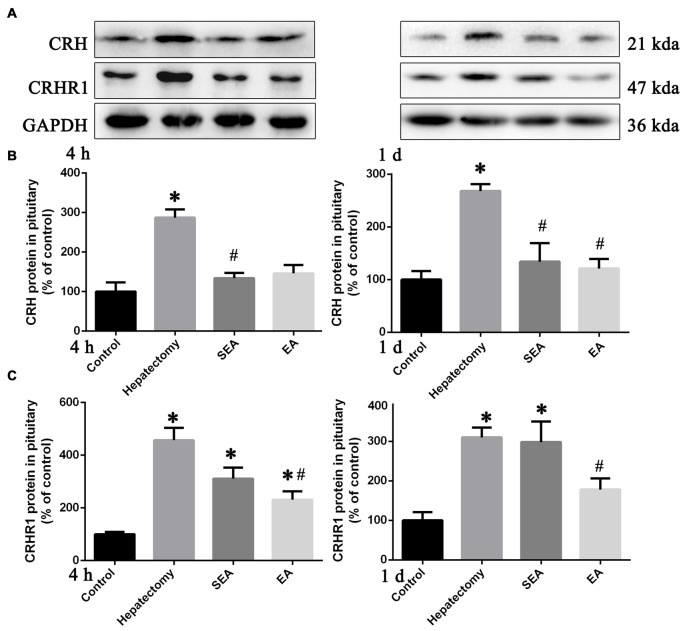
Corticotrophin releasing hormone (CRH) and CRH receptor 1 (CRHR1) protein expression determined by western blot (WB) in the pituitary gland. Representative bands **(A)** and quantification of pituitary CRH **(B)** and CRHR1 **(C)** protein levels in the control, hepatectomy, SEA and EA groups at 4 h and 1 day after hepatectomy. The data are presented as the mean ± SD (*n* = 7) *vs. control group (*p* < 0.05); ^#^vs. hepatectomy group (*p* < 0.05).

Compared with the control group, pituitary CRH protein expression was increased in the hepatectomy group at 4 h (*p* < 0.05) and 1 day (*p* < 0.01) post-surgery (Figures 2A,B). In Comparison with the hepatectomy group, CRH protein expression in the EA group was decreased at 1 day (*p* < 0.05) after hepatectomy (Figures 2A,B). Additionally, the pituitary CRHR1 protein level in the hepatectomy group was up-regulated compared with that in the control group at both 4 h (*p* < 0.05) and 1 day (*p* < 0.05) post-surgery while it was significantly decreased in the EA group compared with the hepatectomy group at both 4 h (*p* < 0.05) and 1 day (*p* < 0.05) after hepatectomy (Figures 2A,C).

**Figure 3:**
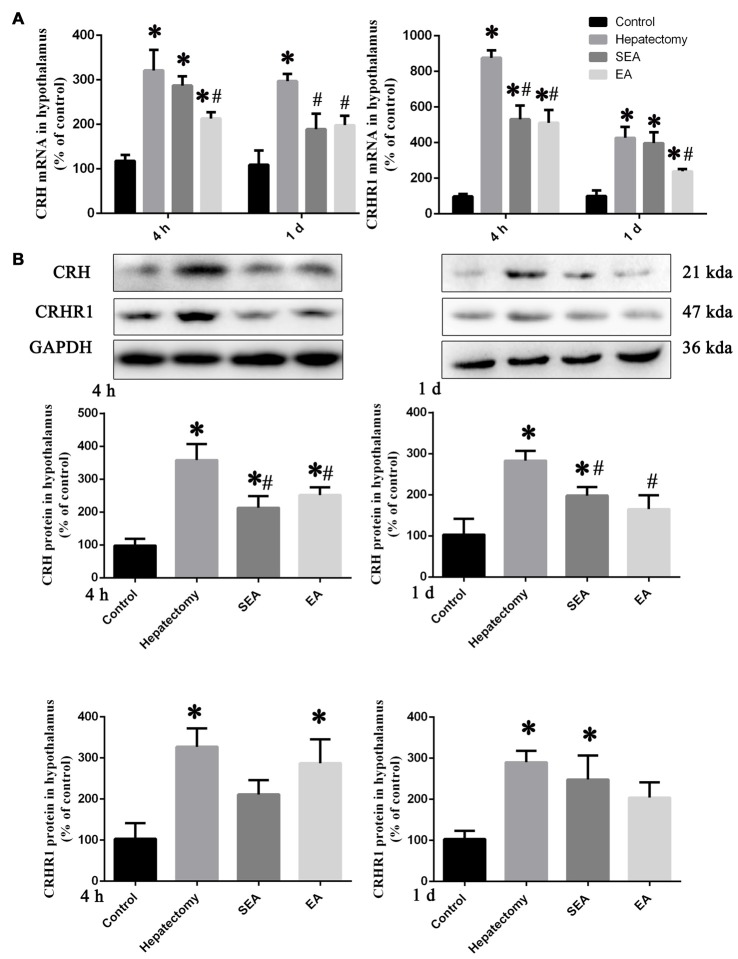
CRH and CRHR1 expression in the hypothalamus. Quantification of the hypothalamus CRH and CRHR1 mRNA levels **(A)** in the control, hepatectomy, SEA and EA groups at 4 h and 1 day after hepatectomy. Representative bands and quantification of hypothalamus CRH and CRHR1 protein levels** (B)** in the control, hepatectomy, SEA and EA groups at 4 h and 1 day after hepatectomy. The data are presented as the mean ± SD (*n* = 7) *vs. control group (*p* < 0.05); ^#^vs. hepatectomy group (*p* < 0.05).

**Figure 4:**
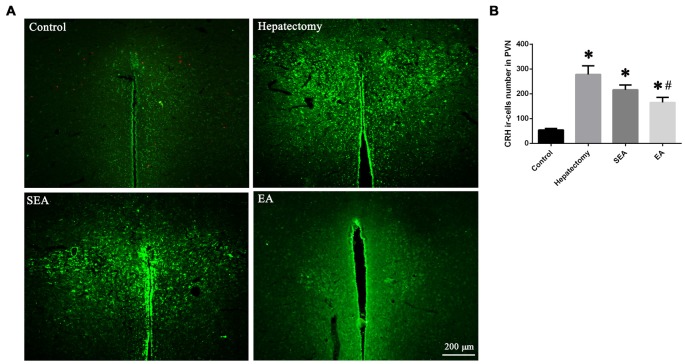
CRH-positive cells in the hypothalamic paraventricular nucleus (PVN). Immunofluorescent assay** (A)** and quantification **(B)** for CRH-positive cells in the hypothalamic PVN among the control, hepatectomy, SEA and EA groups. The data are presented as the mean ± SD (*n* = 5, scale bar = 200 μm) *vs. control group (*p* < 0.05); ^#^vs. hepatectomy group (*p* < 0.05).

The hypothalamus CRH and CRHR1 mRNA levels were increased at 4 h post-surgery in the hepatectomy group (*p* < 0.05), the SEA group (*p* < 0.05) and the EA group (*p* < 0.05) in comparison with the control group. Also, there were significant reduction in the hypothalamus CRH and CRHR1 mRNA expression in the EA group compared with the hepatectomy group at 4 h (*p* < 0.05) and 1 day (*p* < 0.05) after surgery (Figure 3A). Compared with the control group, hypothalamic CRH protein expression in the hepatectomy group (*p* < 0.05), SEA group (*p* < 0.05) was significantly enhanced, while it was decreased in the SEA group (*p* < 0.05) and EA group (*p* < 0.05) compared with the hepatectomy group at 4 h and 1 day after hepatectomy. Besides, compared with the control group, hypothalamic CRHR1 protein expression in the hepatectomy group was up-regulated (*p* < 0.05; Figure 3B). There was a significant increase in the number of PVN CRH-positive neurons in the hepatectomy group compared with that in the control group at 4 h post-surgery and a statistical decrease in the EA group compared with that of the hepatectomy group (*p* < 0.05; Figures 4A,B).

### Intra-PVN Administration of siCRH Reversed HPA Axis Hyperactivity Induced by Hepatectomy

**Figure 5:**
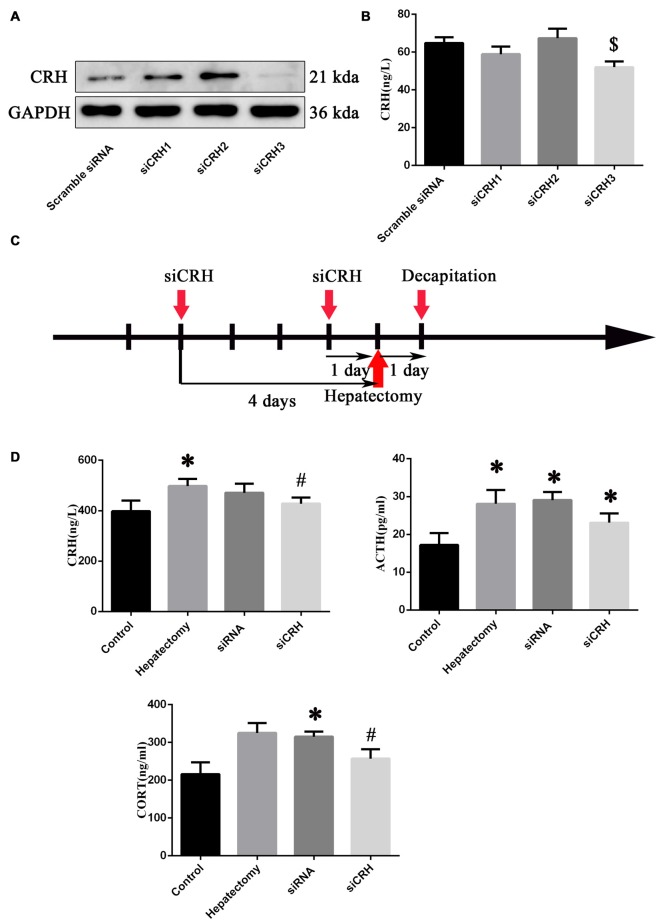
The effect of endogenous CRH knock down on the serum CRH, ACTH and CORT levels of rats at 1 day after hepatectomy. Representative bands **(A)** in primary hypothalamus neurons after culture with siRNAs (*n* = 3) and quantification of the CRH secretion level **(B)** from primary hypothalamus neurons (*n* = 6) ^$^vs. scramble siRNA group (*p* < 0.05). The injection and decapitation time **(C)** of siRNA administration. **(D)** Blood CRH, ACTH and CORT levels among the control, hepatectomy, siRNA and siCRH groups. The data are presented as the mean ± SD (*n* = 5) *vs. control group (*p* < 0.05); ^#^vs. hepatectomy group (*p* < 0.05).

After primary hypothalamus neurons were cultured (Supplementary Figure S3A), we transferred the siRNA into primary hypothalamic neurons. The effective siRNA sequence that could inhibit the expression of CRH protein (*p* < 0.05) and secretion (*p* < 0.05; Figures 5A,B) was: Forward: 5′-GGAUCUCACCUUCCACCUU-dTdT-3′ and Reserve: 3′-dTdT CCUAGAGUGGAAGGUGGAA-5′. Then siCRH or scramble siRNA was injected into PVN nuclei at 4 days and 1 day before surgery, and the peripheral blood was harvested 1 day after surgery (Figure 5C).

It was found that peripheral CRH (*p* < 0.05) and ACTH (*p* < 0.05) in the hepatectomy group compared with the control group were significantly increased. The serum ACTH (*p* < 0.05) and CORT (*p* < 0.05) levels in the siRNA group were increased compared with the control group, while the serum CRH (*p* < 0.01) and CORT (*p* < 0.05) levels in the siCRH group decreased significantly compared with that in the hepatectomy group (Figure 5D).

### CRH Was a Target of miR-142 and miR-376c

**Figure 6:**
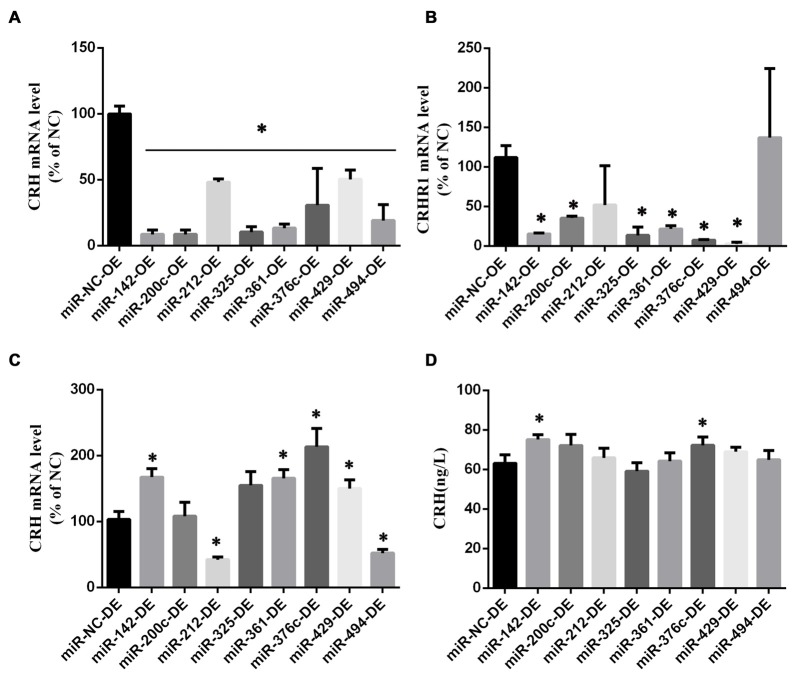
MicroRNAs (miRNA) screening in primary hypothalamus neurons. CRH **(A)** and CRHR1 **(B)** mRNA in primary hypothalamus neurons transfected with miRNA agomir or its scramble control (*n* = 5) *vs. miR-NC-OE group (*p* < 0.05). CRH mRNA level **(C)** and CRH secretion **(D)** in primary hypothalamus neurons transfected with miRNA antagomir or its scramble control. The data are presented as the mean ± SD (*n* = 5) *vs. miR-NC-OE group in **(A,B)** or miR-NC-DE in **(C,D)** (*p* < 0.05).

The miRNAs targeting the 3′-UTR of CRH were determined via bioinformatics analysis^1^. After a transfection efficiency assessment (Supplementary Figure S3B), various miRNA agomirs or antagomirs were transferred into primary cultured hypothalamic neurons (Figure 6, Supplementary Figure S4).

We found that miR-142, miR-200c, miR-212, miR-325, miR-361, miR-376c, miR-429, and miR-494 overexpression could down-regulate the CRH mRNA level (*p* < 0.05; Figure 6A), and there was no difference between these miRNAs and the negative miRNA control (*p* > 0.05) in terms of cell viability (Supplementary Figure S2A). Besides, miR-142, miR-200c, miR-325, miR-361, miR-376c and miR-429 overexpression could decrease the level of CRHR1 mRNA simultaneously (*p* < 0.05; Figure 6B). The decreased expression of the above miRNAs in primary hypothalamus neurons had no effect on cell viability (Supplementary Figure S2A), and the decreased expression of the miR-142, miR-361, miR-376c and miR-429 could increase CRH mRNA (*p* < 0.05; Figure 6C). Also, miR-142 and miR-376c down expression could up-regulate CRH secretion simultaneously (*p* < 0.05; Figure 6D).

**Figure 7:**
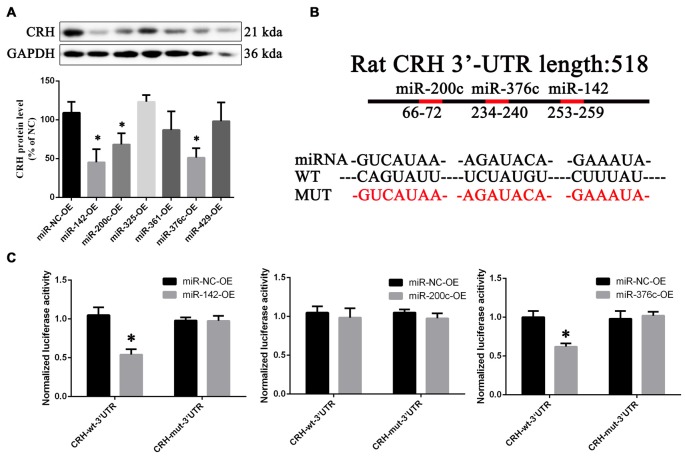
CRH is a target of miR-142 and miR-376c. CRH protein expression **(A)** in primary hypothalamus neurons transfected with miR-142, miR-200c, miR-325, miR-361, miR-376c and miR-429 agomir or the negative control. The data are presented as the mean ± SD (*n* = 5) *vs. miR-NC-OE group (*p* < 0.05). The binding site** (B)** of miR-142, miR-200c and miR-376c on the 3′-untranslatedregions (3′ UTR) of CRH and their mutant plasmid. The luciferase activity **(C)** of the miR-142, miR-200c, miR-376c onto the 3′ UTR of CRH. The data are presented as the mean ± SD (*n* = 4) *vs. miR-NC-OE group (*p* < 0.05).

Compared with overexpression of the scramble miRNA, miR-142, miR-200c and miR-376c could down-regulate CRH protein in primary cultured hypothalamus neurons (*p* < 0.05; Figure 7A). In the dual luciferase reporter system, miR-142 (0.55 ± 0.037) and miR-376c (0.6 ± 0.045) over-expression significantly reduced the fluorescein enzyme activity (*p* < 0.01), whereas miR-200c had no change (*p* > 0.05; Figures 7B,C).

### Hypothalamic miR-142 and miR-376c Levels after Hepatectomy

**Figure 8:**
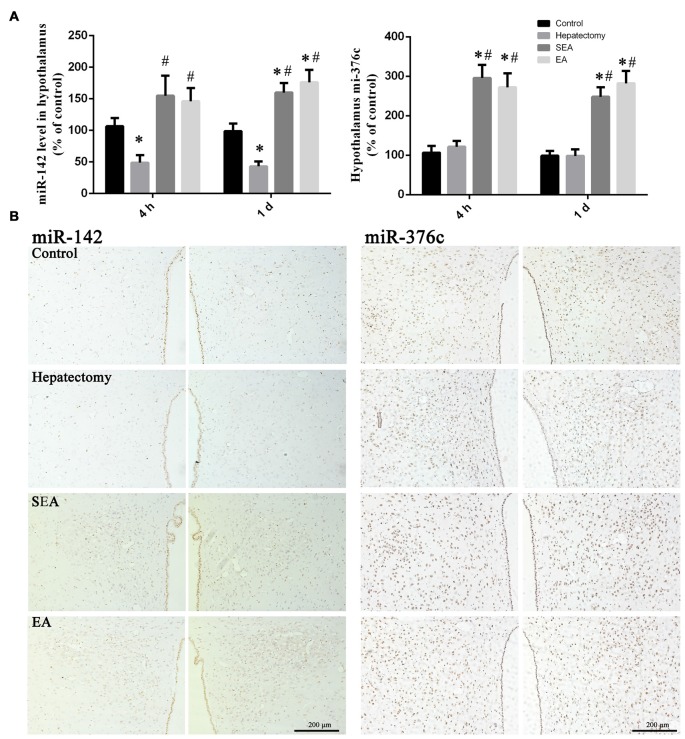
miR-142 and miR-376c expression in the hypothalamus. Quantification of the expression levels of miR-142 and miR-376c among the control, hepatectomy, SEA and EA groups at 4 h and 1 day after hepatectomy **(A)**. The data are presented as the mean ± SD (*n* = 7) *vs. control group (*p* < 0.05); ^#^vs. hepatectomy group (*p* < 0.05). Detection of miR-142 and miR-376c in the PVN among groups at 1 day after hepatectomy **(B)**. Scale bar = 200 μm (*n* = 5).

Compared with the control group, the hypothalamus miR-142 level in the hepatectomy group was decreased at 4 h (*p* < 0.01) and 1 day (*p* < 0.01) after hepatectomy. In comparison with the hepatectomy group, the hypothalamus miR-142 level in the SEA group (*p* < 0.05) and the EA group (*p* < 0.01) was increased at 4 h after surgery. At 1 day after hepatectomy, the hypothalamus miR-142 level in the SEA group (*p* < 0.01) and the EA group (*p* < 0.01) was increased compared with that in the hepatectomy group (Figure 8A). Meanwhile, PVN miR-142 was decreased compared with the control group in the hepatectomy group (*p* < 0.05), and compared with the hepatectomy group (Figure 8B), it was increased in the EA group (*p* < 0.05).

There was no difference between the hepatectomy group and the control group in terms of the hypothalamus miR-376c expression at 4 h (*p* > 0.05) and 1 day (*p* > 0.05) after surgery. Compared with the control group, the hypothalamus miR-376c expression in the SEA group (*p* < 0.01) and the EA group (*p* < 0.01) was increased at 4 h and 1 day after hepatectomy. Besides, the hypothalamus miR-376c expression in the SEA group (*p* < 0.01) and EA group (*p* < 0.01) was increased at 4 h and 1 day after hepatectomy compared with the hepatectomy group (Figure 8A). There was no difference between the control and hepatectomy group in the terms of PVN miR-376c level (*p* > 0.05; Figure 8B). In addition, there was no difference of miR-142 (*p* > 0.05) or miR-376c (*p* > 0.05) expression in the ventromedial hypothalamic nucleus (VMH), periventricular hypothalamic nucleus (Pe) and arcuate hypothalamic nucleus (Arc) among groups (Supplementary Figure S5).

### PVN miR-142 or miR-376c Overexpression Alleviated HPA Axis Hyperactivity Induced by Hepatectomy

**Figure 9:**
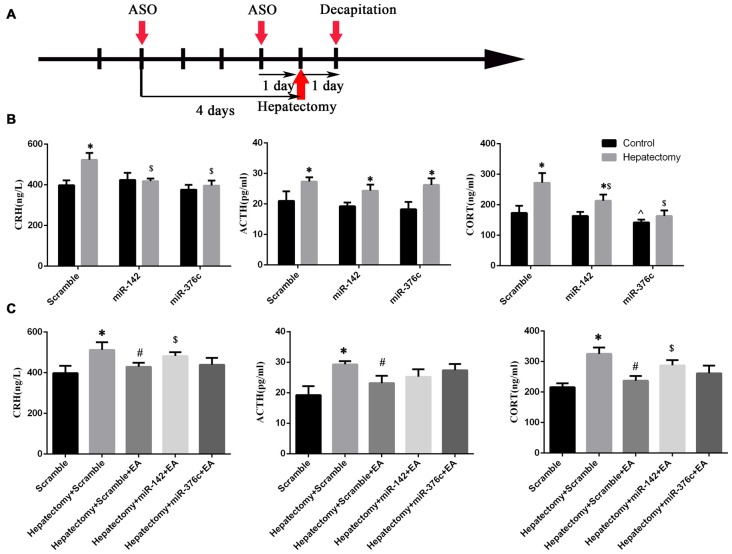
The effect of hypothalamus miR-142 and miR-376c on the hypothalamus pituitary adrenal (HPA) axis during hepatectomy. The injection time **(A)** of the ASO (miRNA agomir or antagomir) and their scramble control was 4 days and 1 day before hepatectomy. The effect of the overexpression of miR-142 or miR-376c on the change in serum CRH, ACTH and CORT levels **(B)**. The data are presented as the mean ± SD (*n* = 6) *vs. control group (*p* < 0.05); ^$^vs. hepatectomy+scramble group (*p* < 0.05); ^vs. control+scramble group (*p* < 0.05). The effect of the down-regulation of miR-142 or miR-376c on the change in serum CRH, ACTH and CORT levels **(C)**. The data are presented as the mean ± SD (*n* = 6) *vs. scramble group (*p* < 0.05); ^#^vs. hepatectomy+scramble group (*p* < 0.05); ^#^vs. hepatectomy+scramble group (*p* < 0.05); ^$^vs. hepatectomy+scramble+EA group (*p* < 0.05).

After the installation of the rat brain stereotaxic trace delivery system, the miRNA agomir or scramble miRNA was injected into PVN nuclei at 4 days and 1 day before hepatectomy (Figure 9A), then peripheral blood was harvested at 1 day after hepatectomy. Serum CRH (*p* < 0.01), ACTH (*p* < 0.05) and CORT (*p* < 0.01) were upregulated in the scramble+hepatectomy group compared with the scramble+control group. The serum ACTH (*p* < 0.01) and CORT (*p* < 0.01) levels increased in the miR-142+hepatectomy group compared with those in the miR-142+control group. Additionally, the serum ACTH (*p* < 0.01) and CORT (*p* < 0.05) levels decreased in the miR-142+hepatectomy group compared with those in the scramble+hepatectomy group. It could be observed that compared with the miR-376c+control group, the serum ACTH level was up-regulated in the miR-376c+hepatectomy group (*p* < 0.01). Also, the peripheral serum CRH (*p* < 0.01) and CORT (*p* < 0.05) levels in the miR-376c+hepatectomy group were down-regulated in comparison with the scramble+ hepatectomy group. In addition, the serum CORT level in the miR-376c+control group was decreased in comparison with that in the rats of the scramble +control group (*p* < 0.05; Figure 9B).

### The Regulatory Effect of EA on HPA Axis Hyperactivity in the Hepatectomy Rats Was Blocked by Intra-PVN Administration of miR-142 Antagomir

After the installation of the rat brain stereotaxic trace delivery system, the miRNA antagomir or scramble miRNA was injected into PVN nuclei at 4 days and 1 day before surgery (Figure 9A). Decreased expression of miR-142 or miR-376c via antagomir in the PVN of rats was seen compared with the rats in the scramble group. Also, the peripheral blood CRH (*p* < 0.05), ACTH (*p* < 0.05) and CORT (*p* < 0.01) expression levels were increased in rats of the scramble+hepatectomy group. Serum CRH (*p* < 0.05), ACTH (*p* < 0.05) and CORT (*p* < 0.01) expression levels were decreased in the hepatectomy+scramble+EA group compared with those in the hepatectomy+scramble group; compared with the hepatectomy+scramble+EA group, blood CRH (*p* < 0.05) and CORT (*p* < 0.01) levels were increased in the hepatectomy+miR-142+EA group (Figure 9C).

## Discussion

Surgery or severe trauma caused endocrine disturbance and immunopression; thus either may prompt vulnerability in psychopathology and increase risk for negative health outcomes (Cacciaglia et al., 2017). Hepatectomy induced inflammatory, immune system dysfunction (Shimada et al., 1996) and neurotransmitter secretion. Our previous study also demonstrated an HPA axis disorder in response to hepatectomy (Zhu et al., 2016). Taking this into account, we decided to further elucidate the underlying mechanism related to the surgery-induced HPA axis in a hepatectomy model.

GC expression was the primary index to evaluate the function of the HPA axis (Khan et al., 2011). GC was vital in maintaining the HPA axis equilibrium, and excessive GC level led to immunosuppression. In the present study, serum GC was highly expressed after surgical trauma and attenuated by the application of EA. This regulation strongly supported the notion that EA could alleviate the hyperactivity of the HPA axis induced by hepatectomy.

Stressful stimulus information, such as pain, hemorrhage, and hypoxia induced by trauma, was conducted to the PVN (Aguilera and Liu, 2012). Norepinephrine (NE), glutamate, gamma Aminobutyric acid (GABA), 5-Hydroxytryptamine (5-HT), interleukin-1β (IL-1β) and neuropeptide (NPY) had been demonstrated to influence CRH and the HPA axis function (Aguilera and Liu, 2012). Many stressors could activate the brain stem noradrenergic, stimulate the noradrenaline release in the PVN, and regulate the CRH expression (Haisenleder, 2000). Glutamate played an important role in coordination of HPA axis output through excitatory signaling via ionotropic glutamate receptors and inhibited signaling via group I metabotropic receptors in the PVN (Evanson and Herman, 2015). The role of GABAergic inhibition in the PVN on the CRH neurons and its impact on the HPA axis has been reported (Lee et al., 2014). 5-HT modulated hypothalamus CRH and thus serum CORT via its receptors (Mikkelsen et al., 2004). IL-1β administration increased CRH mRNA in the PVN (Hsieh et al., 2010), as well as plasma ACTH and CORT level (Gadek-Michalska et al., 2008). PVN NPY increase CRH synthesis and circulatory ACTH and CORT (Wahlestedt et al., 1987).

EA was proved to regulate HPA axis function in chronic unpredictable mild stress (CUMS; Le et al., 2016), cold stress (Eshkevari et al., 2013), CORT-induced depression model (Lee et al., 2009), murine asthma (Wei et al., 2017) and the hepatectomy model. Meanwhile, EA could increase hippocampus 5-HT to improve depression in CUMS model and adrenal NPY with no significance noted in circulating NE in cold stress model and serum inflammation in murine asthma model. EA was verified to regulate NE (Fornes et al., 2016), glutamate (Zeng et al., 2016), GABA (Fu and Longhurst, 2009), 5-HT (Cui et al., 2016), NPY (Tian et al., 2006) and IL-1β (Qin et al., 2013), but no evidence shown that EA affect HPA axis function via NE, glutamate, GABA, 5-HT, NPY or IL-1β. Different types of stressors, physical or psychological, acute or chronic cause various stress reactions. The expression pattern and function of them in the hepatectomy should be elucidated in future studies.

Hypothalamus parvocellular-secreted CRH is the initial part of the HPA axis. It reacts to its receptor, CRHR1, in the corticotrops to modulate ACTH synthesis and secretion. We thus detected the pituitary CRHR1 expression and the hypothalamus CRH mRNA and protein levels. It was found that both pituitary CRHR1 and hypothalamus CRH were increased at 4 h and 1 day after surgery. The decreased CRH expression in the hypothalamus could down-regulate the serum GC level induced by trauma, which indicated the crucial role of CRH in the HPA axis during hepatectomy. CRHR1 and CRH elevation induced by hepatectomy was alleviated by EA. The same result was also found in cold stress (Eshkevari et al., 2013), chronic heterotypic stress (Zhao et al., 2017) and CUMS (Le et al., 2016), which demonstrated a role for EA in the HPA axis regulation.

To date, EA has been proven to inhibit microglia activation (Tang et al., 2016), attenuate inflammation (Wang et al., 2009), reduce oxidative stress (Chen Y. et al., 2016), and maintain homeostasis (Li M. et al., 2014), which are all stress-related. Interestingly, study had utilized a pretreatment of EA at ST 36 as an acupoint (Chen Y. et al., 2016). A previous study illustrated that ST 36 stimulation was related to the integration of cerebrocerebellar and limbic system (Hui et al., 2005). EA at SP6 and ST36 with 2/15 Hz could produced a better analgesic effect in rat model of laparotomy (Feng et al., 2012). Our previous study showed that EA at ST36 and SP6 could improve the hyperactivity of the HPA axis in hepatectomy rats (Zhu et al., 2016). Thus, we assessed the EA application in this study.

Advances in the last decade have identified miRNA regulation as an important effector in CNS development and disease. Given that the regulation role of miRNAs depends on binding to the 3′-UTR of its target mRNA, which lays the foundation for prognosis, the target of miRNAs is on an appointed mRNA, such as CRH. Targetscan and miRBASE were used for bioinformatic analysis in the present study, and we screened the candidate miRNAs in primary cultured hypothalamic neurons. Finally, a dual luciferase reporter gene assay verified that CRH was a target of miR-142 and miR-376c. Furthermore, the level of hypothalamus miR-142 was decreased in the hepatectomy rats, which indicated that miR-142 was a potential miRNA in regulating the HPA axis during severe trauma. Moreover, miRNAs were reported to extensively respond to various therapeutic interventions; thus, we focused on their role in the attenuation of the HPA axis hyperactivity by EA.

Recent research showed that hypothalamus let-7, miR-148a, miR-124, miR-107 and miR-370 were confirmed to be related to EA tolerance (Cui et al., 2017). EA pretreatment had a protective effect on ischemia/reperfusion injury via miR-214 (Liu et al., 2014) and miR-124 (Chen S. H. et al., 2016). Microarray assay showed that acupuncture at a specific acupoint increased let-7b, miR-339, miR-223, miR-145, miR-451, miR-193, miR-378 and miR-423 and decreased let-7a, miR-9, miR-128 and miR-132 while a lack of acupuncture failed to affect the level of these miRNAs in the medulla of spontaneously hypertensive rats (Wang et al., 2015). This regulation strongly supported the involvement of miRNAs in EA modulation. We found that EA could increase the expression of both miR-142 and miR-376c in the hypothalamus, and miR-142 down-regulation could inactivate the role of EA in HPA axis regulation during trauma. However, the underlying mechanism for hypothalamus miR-142 and miR-376c expression regulated by EA still needs to be further explored.

In addition, it should be noted that miRNAs may be expressed differently in various cell types, and could modulate target mRNA directly and indirectly. Besides, one miRNA may have more than one target mRNA, it is hard to evaluate the interaction among its target mRNAs. Much work is required at some additional levels, further studies are needed to characterize the cell type differences and the interaction of its target mRNAs.

## Conclusion

EA can improve surgical trauma-induced HPA axis hyperactivity via reducing the hypothalamic CRH levels. Hypothalamic miR-142 and miR-376c could modulate the function of the HPA axis by targeting CRH. Partial hepatectomy induced the downregulation of hypothalamic miR-142 levels, and the effect of EA in adjusting the CRH level and attenuating the surgery-induced hyperactivity of the HPA axis mainly by up-regulating the expression of miR-142 and miR-376c occurred within the hypothalamus.

## Author Contributions

ZT contributed to experimental design, data interpretation and editing of manuscript. JZ performed surgery, plasmid construction and luciferase assay, cell culture, cell experiment and wrote the manuscript. ZC performed RT-PCR, WB, ELISA and ISH experiment. ZM performed the surgery, IF and data analysis. MJ, GW, HG contributed to the EA experiment, data analysis and interpretation. MZ contributed to the ELISA.

## Conflict of Interest Statement

The authors declare that the research was conducted in the absence of any commercial or financial relationships that could be construed as a potential conflict of interest.

## Footnotes

^1^http://www.mirbase.org/index.shtml; http://www.microrna.org/microrna/home.do; and http://www.targetscan.org/
