# Supplementary material for: Electroacupuncture Alleviates Surgical Trauma-Induced Hypothalamus Pituitary Adrenal Axis Hyperactivity Via microRNA-142
Source: Front Mol Neurosci. 2017 Sep 27;10:308. doi: 10.3389/fnmol.2017.00308 (PMC5623716; doi:10.3389/fnmol.2017.00308)
Supplement: Supplementary file 1 [file Data_Sheet_1.docx]

Table 1. The siRNA candidate sequences for inhibiting CRH were designed as follows:

| siRNA candidate | | sequence |
| --- | --- | --- |
| 1 | Forward: | 5‘ GCCGUUGAAUUUCUUGCAA dTdT 3‘ |
|  | Reverse: | 3‘ dTdT CGGCAACUUAAAGAACGUU 5‘ |
| 2 | Forward: | 5‘ GCAUGGGUGAAGAAUACUU dTdT 3‘ |
|  | Reverse: | 3‘ dTdT CGUACCCACUUCUUAUGAA 5‘ |
| 3 | Forward: | 5‘ GGAUCUCACCUUCCACCUU dTdT 3‘ |
|  | Reverse: | 3‘ dTdT CCUAGAGUGGAAGGUGGAA 5‘ |

Table 2. The primers used to generate wild type and mutant CRH 3’-UTR were as follows:

| Rat-CRH-3’UTR | | Sequence | Amplification length |
| --- | --- | --- | --- |
| Wt | Forward | GCGCTCGAGGAAATGAAATGTTGCGCTTG | 1,172 bp |
|  | Reverse | AATGCGGCCGCGACACAACCAAATTGAC |  |
| miR-142- mut | F245 | ATAAGTGTGAAATAAGCGATATCTTAAAGAAAAT | 478 bp |
|  | R266 | ATATCGCTTATTTCACACTTATTTGCACATAG |  |
| miR-200c- mut | F58 | TTAAAACAGTCATAACTGTACCATACTGCAGCT | 1,172 bp |
|  | R80 | TGGTACAGTTATGACTGTTTTAAATTTTTTATT |  |
| miR-376c- mut | F226 | GCTTAGTTAGATACAGCAAATAAGTGTCTTTAT | 1,172 bp |
|  | R248 | TTATTTGCTGTATCTAACTAAGCGTGAACAATA |  |

Table 3. Primers used for Real-Time PCR.

| Gene symbol | Primer | |
| --- | --- | --- |
| CRH | Forward: | CTC TCT GGA TCT CAC CTT CCA C |
|  | Reverse: | CTA AAT GCA GAA TCG TTT TGG C |
| CRHR1 | Forward: | TGG AAC CTC ATC TCG GCT TT |
|  | Reverse: | GTG AGC TGG ACC ACA AAC CA |
| GAPDH | Forward: | GTA TGA CTC TAC CCA CGG CAA GT |
|  | Reverse: | TTC CCG TTG ATG ACC AGC TT |


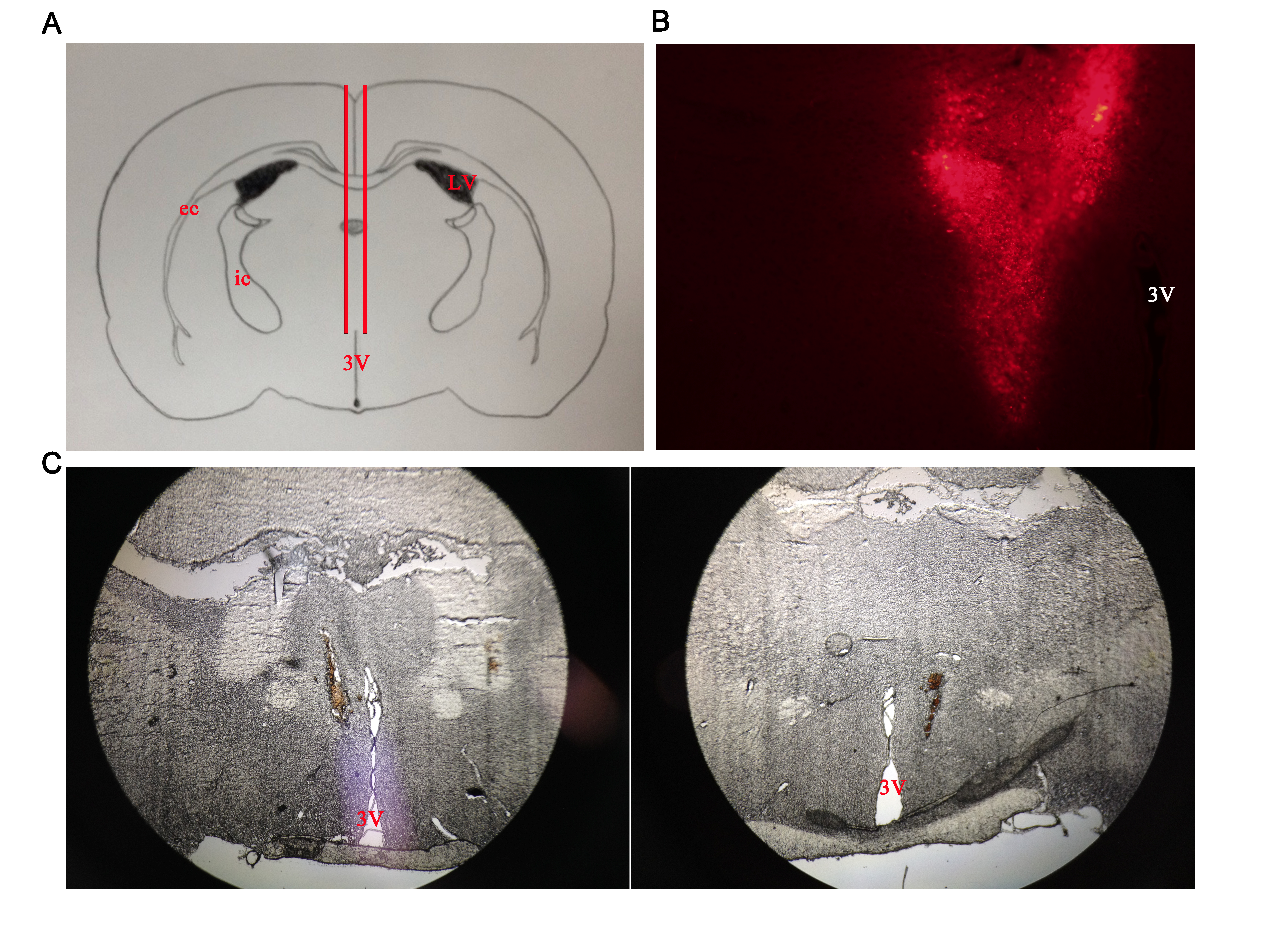


**Fig. S1** The injection site. The diagram of injection sites (A) and injected cy-3 labelled siRNA (B) in the PVN. The injection site with Injected ink (C). 3V: third ventricle; LV: lateral ventricle; ic: internal capsule; ec: external capsule.


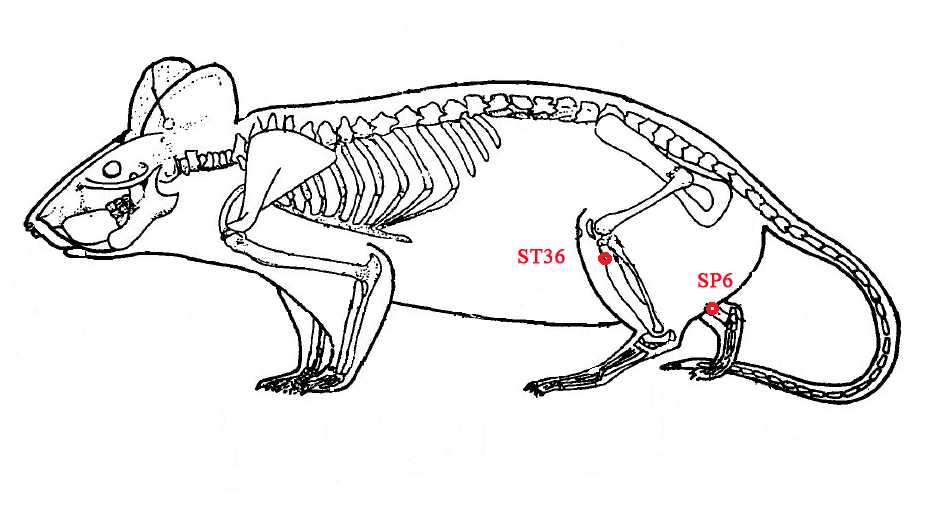


**Fig. S2** The location of ST36 and SP6 in rat. ST36 (Zu San Li), located in the posterolateral knee joint, approximately 0.5 cm below the capitulum fibulae; SP6 (San Yin Jiao), at the superior border of the media malleolus, between the posterior border of the tibia and anterior border of the Achilles tendon.


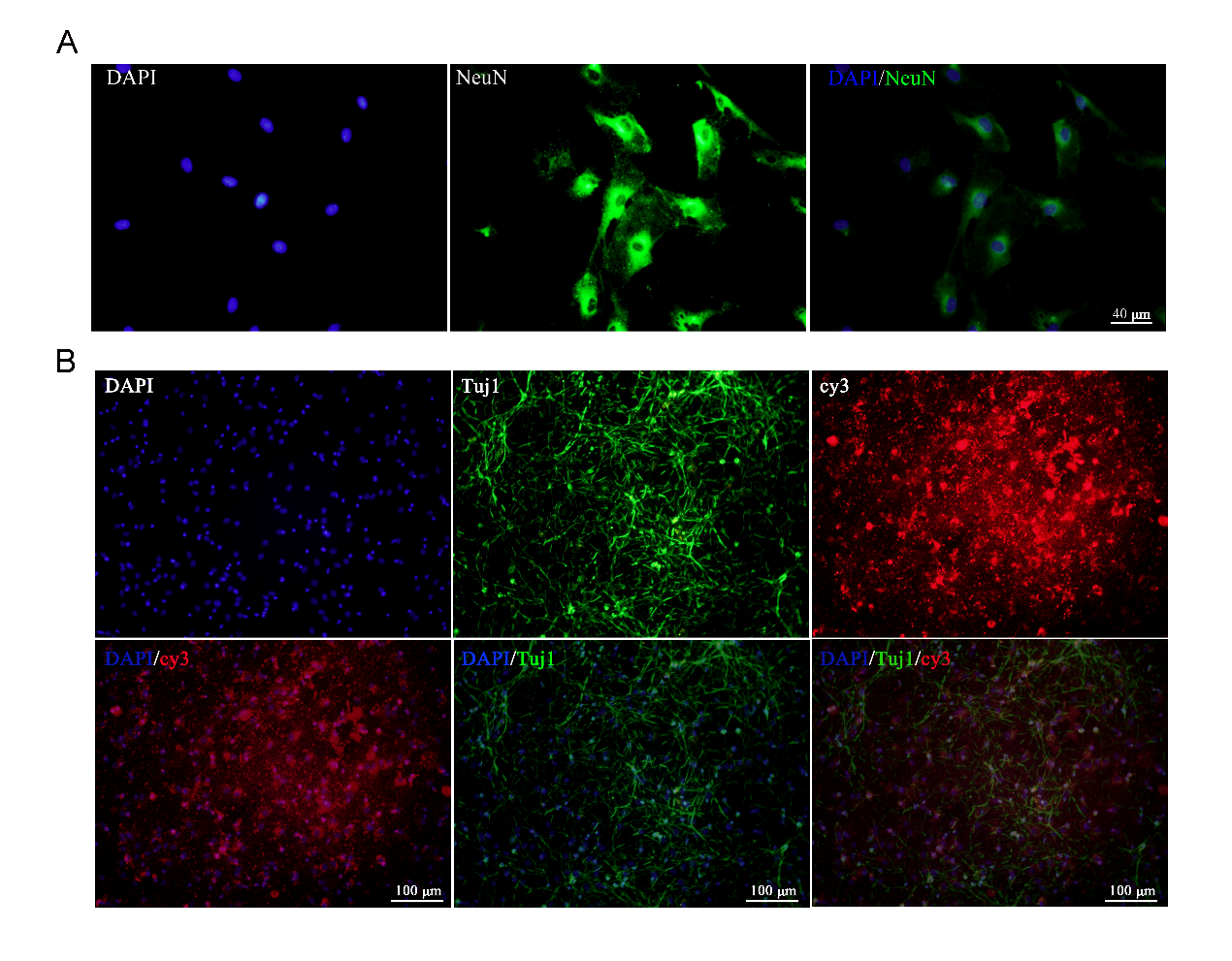


**Fig. S3** Immunofluorescent assay for the identification of primary hypothalamic neurons and cy-3 marked miRNA scramble control transfection efficiency. NeuN-positive cells in the cultured primary hypothalamus cells (A), scale bar=40 μm. The transfection efficiency of the cy-3 marked miRNA scramble control (B), scale bar=100 μm.


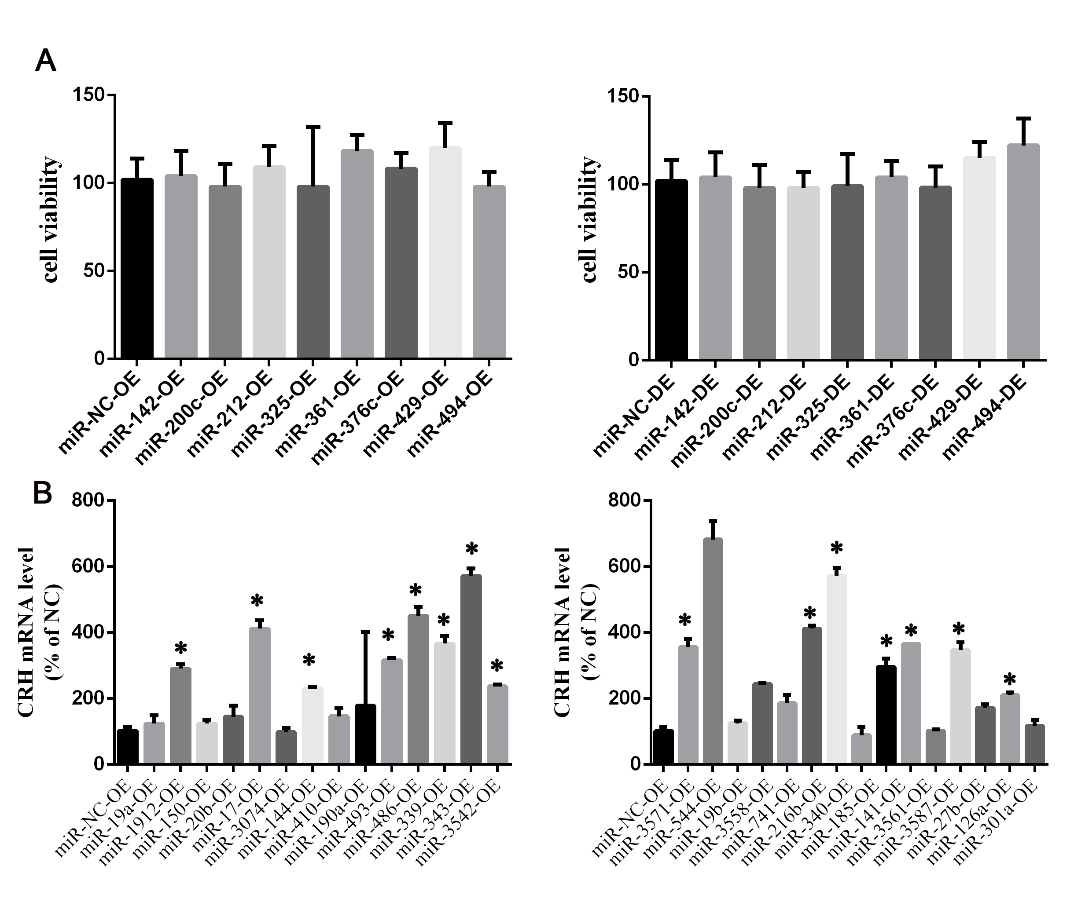


**Fig. S4** The overexpression and down-regulation of miRNA in primary hypothalamic neurons. The cell viability (A) of the overexpressed miRNAs and their downregulation in primary hypothalamic neurons. The CRH mRNA level (B) in primary hypothalamic neurons after overexpression of miRNAs predicted by bioinformatics. The data are presented as the mean ± SD (n=4). *, versus miR-NC-OE group (p < 0.05).


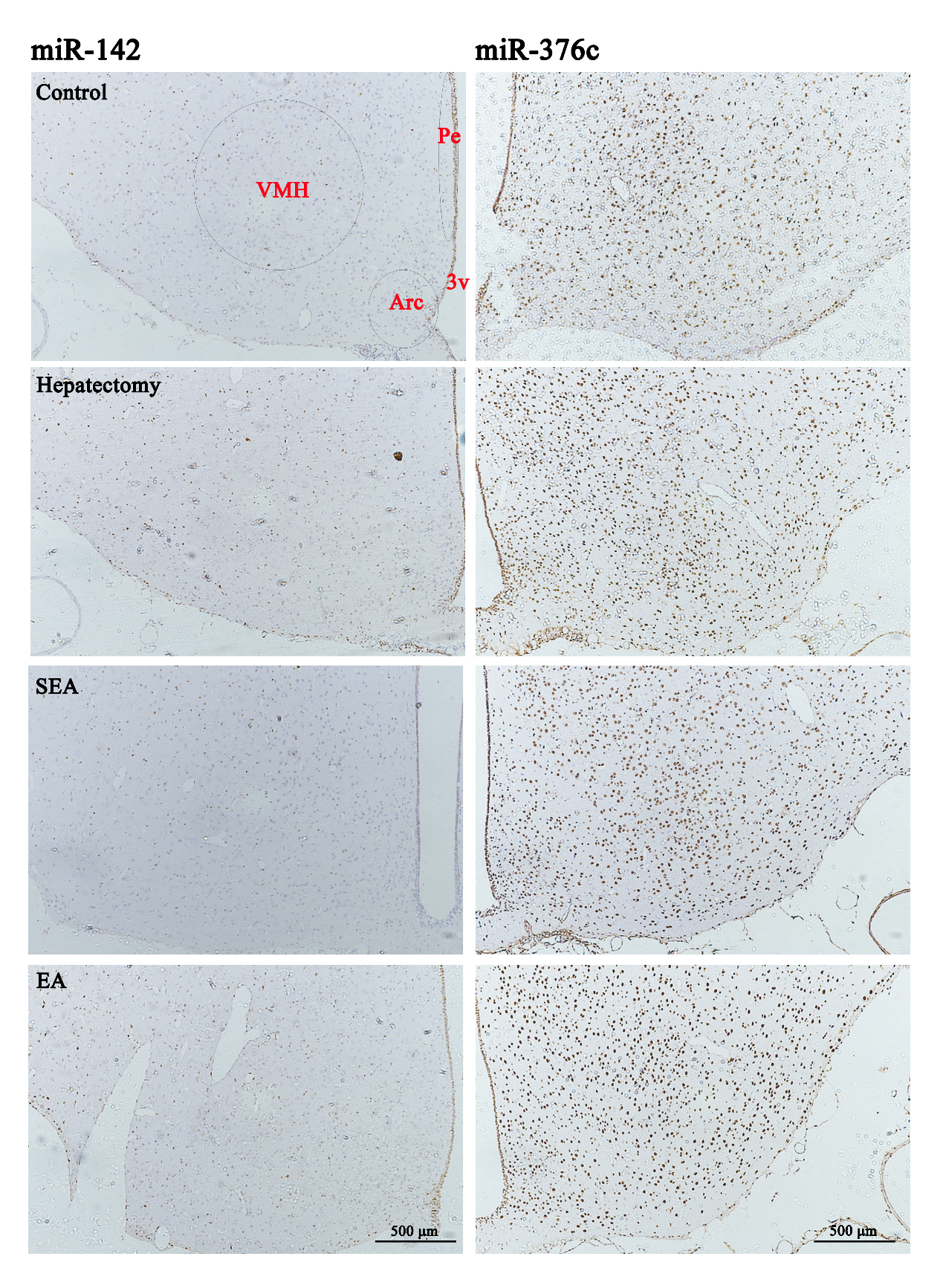
**Fig. S5** miR-142 and miR-376c expression in the arcuate hypothalamic nucleus (Arc), ventromedial hypothalamic nucleus (VMH) and periventricular hypothalamic nucleus (Pe) among the control, hepatectomy, SEA and EA groups at 1 day after hepatectomy. 3V: third ventricle; Scale bar= 500 μm (n=5).
